# Autophagy Machinery as a Promising Therapeutic Target in Endometrial Cancer

**DOI:** 10.3389/fonc.2019.01326

**Published:** 2019-11-29

**Authors:** Stephanie I. Nuñez-Olvera, Dolores Gallardo-Rincón, Jonathan Puente-Rivera, Yarely M. Salinas-Vera, Laurence A. Marchat, Raúl Morales-Villegas, César López-Camarillo

**Affiliations:** ^1^Posgrado en Ciencias Genómicas, Universidad Autónoma de la Ciudad de México, Mexico City, Mexico; ^2^Laboratorio de Medicina Translacional, Instituto Nacional de Cancerología, Mexico City, Mexico; ^3^Departamento de Ecología Funcional, Instituto de Ecología, Universidad Nacional Autónoma de México, Mexico City, Mexico; ^4^Programa en Biomedicina Molecular y Red de Biotecnología, Instituto Politécnico Nacional, Mexico City, Mexico; ^5^Coordinación Académica Huasteca del Sur, Universidad Autónoma de San Luis Potosí, San Luis Potosí, Mexico

**Keywords:** endometrial cancer, autophagy, AKT/mTOR pathway, microRNAs, therapy

## Abstract

Endometrial cancer is the fourth most frequent neoplasia for women worldwide, and over the past two decades it incidence has increased. The most common histological type of endometrial cancer is endometrioid adenocarcinoma, also known as type 1 endometrial cancer. Endometrioid endometrial cancer is associated with diverse epidemiological risk factors including estrogen use, obesity, diabetes, cigarette smoking, null parity, early menarche, and late menopause. Clinical effectiveness of chemotherapy is variable, indicating that novel molecular therapies against specific cellular processes associated to cell survival and resistance to therapy, such as autophagy, urged to ameliorate the rates of success in endometrial cancer treatment. Autophagy (also known as macroautophagy) is a specialized mechanism that maintains cell homeostasis which is activated in response to cellular stressors including nutrients deprivation, amino acids starvation, hypoxia, and metabolic stress to prolong cell survival via lysosomal degradation of cytoplasmic macromolecules and organelles. However, in human cancer cells, autophagy has a controversial function due to its dual role as self-protective or apoptotic. Conventional antitumor therapies including hormones, chemotherapy and ionizing radiation, may activate autophagy as a pro-survival tumor response contributing to treatment resistance. Intriguingly, if autophagy continues above reversibility of cell viability, autophagy can result in apoptosis of tumor cells. Here, we have reviewed the mechanisms of autophagy described in endometrial cancers, including the role of PI3K/AKT/mTOR, AMPK-mTOR, and p53 signaling pathways that trigger or inhibit the process and thus representing potential molecular targets in therapeutic clinical approaches. In addition, we discussed the recent findings indicating that autophagy can be modulated using repurposing drugs which may leads to faster experimentation and validation, as well as more easy access of the medications to patients. Finally, the promising role of dietary compounds and microRNAs in autophagy modulation is also discussed. In conclusion, although the research about autophagy is scarce but ongoing in endometrial cancer, the actual findings highlight the promising usefulness of novel molecules for directing targeted therapies.

## Endometrial Cancer

Endometrial cancer is the fourth most frequent neoplasia for women worldwide (1). The incidence of endometrial cancer has increased around than 20%, and currently, ~1 in 37 women will develop endometrial cancer during their lifetime (2, 3). The age of onset of endometrial cancer is typically in postmenopausal women, although in the last decade the incidence in young women has dramatically increased as a result of earlier-onset obesity and hyperinsulinemia (4). The mean age at cancer diagnosis is 63 years. Endometrial cancer is originated in the uterine epithelium and can be classified into diverse histological subtypes: (i) endometrioid endometrial cancer (EEC, or Type I), (ii) serous endometrial cancer (SEC, or Type II), (iii) clear cell endometrial cancer (CCEC, or Type II), and (iv) mixed endometrial cancer and uterine carcinoma (USC), which have different clinical and molecular features, as well as prognosis and therapeutic regimen (5). Type I tumors are the most frequent subtype representing about 70% of diagnosed cases, they are low grade and associated to estrogen stimulation, whereas type II tumors are generally high grade, estrogen-independent, less common, clinically aggressive, metastatic, and exhibit a increased risk of relapse after chemotherapy. Type II tumors accounts for 10% of endometrial cancers, but it's related with poor prognosis and 40% deaths (2, 5). However, a large study of the Epidemiology of Endometrial Cancer Consortium USA suggested that the etiology of type II tumors may not be completely estrogen independent (6). Endometrioid endometrial cancer is associated with diverse epidemiological risk factors including unopposed estrogen use, obesity, diabetes, cigarette smoking, null parity, early menarche and late menopause (6, 7). Increased risk for endometrial cancer development is associated in less extend with: (i) Lynch syndrome (2–6% of ECs) caused by monoallelic germline mutation in MLH1, MSH2, MSH6, or PMS2 mismatch repair genes (8), (ii) polymerase proofreading-associated polyposis attributed to germline mutations of the DNA polymerase-delta 1 (POLD1) or DNA polymerase-epsilon (POLE) (9), and Cowden syndrome caused by tumor suppressor PTEN mutations (10). Combinatory therapy with carboplatin and paclitaxel is the main front-line chemotherapy in endometrial cancer (11). These agents are characterized by its capacity to generate DNA damage and bock the proper polymerization of actin microtubules in cytoskeleton, respectively (12). However, its clinical effectiveness is variable, indicating that novel molecular therapies against specific cellular processes associated to cell survival and therapy resistance, such as autophagy, urged to ameliorate the rates of success in endometrial cancer therapies.

## Autophagy: Mechanisms and Functions

Autophagy (also known as macroautophagy) is a highly specialized and evolutionarily conserved process that maintains cell homeostasis (13). Autophagy is activated through a specific transcriptional program (see below) in response to continuous cellular stressors including nutrients deprivation, amino acids starvation, hypoxia, and metabolic stress (14). This self-eating cellular mechanism prolongs survival under diverse stressors via lysosomal degradation of cytoplasmic macromolecules and organelles (15). The autophagic flux is characterized by: (i) the enclose of intracellular cytoplasmic components, macromolecular complexes, long-lived soluble and aggregated proteins, and cellular organelles in vesicles dubbed as autophagosomes, (ii) the degradation of cytoplasmic constituents by fusion of autophagosomes in the lysosomes, and (iii) the reuse of ATP and other molecules for cell biosynthesis. Two main features distinguish the catabolic autophagy: (i) basal autophagy which carried out a key function in homeostasis by reducing the pool of lasting proteins and organelles. Normal cells activate basal autophagy in response to diverse stressors as a temporary cell survival mechanism (16), (ii) induced autophagy in disease conditions, for instance in human cancer cells autophagy has a controversial and complicated function due to its dual role as self-protective or apoptotic. It has been reported that antitumor therapies including hormones, chemotherapy and ionizing radiation, may activate autophagy as a pro-survival tumor response contributing to treatment resistance. Intriguingly, if autophagy continues beyond reversibility of cell viability, autophagy can result in apoptosis of tumor cells (17). The dual role of autophagy during carcinogenesis difficult the efforts to understand how to modulate it to achieve successful treatments, suggesting that genetic mutational background and tumor cell type specific knowledge should be required (18).

In mammalian cells, autophagy depends on the function of the core autophagy proteins (ATG) which initiate the assembly of the omegasome at reticulum endoplasmic, followed by the phagophore formation and later the autophagosomes which fused with lysosomes (19). Briefly, the molecular autophagic pathway can be divided in three steps: (i) after a stimulus, such as nutrients starvation, the ATG proteins are assembled into functional ULK 1/2 and PtdIns3P protein complexes, which are engaged to inner membranes to begin the formation of the omegasome (20). The ULK complex is formed by diverse proteins including ULK1, ULK2, ATG13, RBCC1/FIP200, and ATG101; whereas the class III lipid kinase complex I is formed by ATG14, BECN1/Beclin 1, PIK3R4/p150, and PIK3C3/VPS34. Initiation of autophagy begins when the ULK complex is activated by AMPK kinase (or repressed by mTORC1) at the endoplasmic reticulum membranes that have been previously marked by ATG9 resulting in the formation of the omegasome. Remarkably, there are evidences suggesting that Golgi apparatus is important for the production of ATG9-containing vesicles (named the ATG compartment visualized as small vesicles and tubules) that nucleate the membranous structures shaping the omegasome in order to merge with the phagophore (21). (ii) Then, the phosphatidylinositol 3-kinase (PtdIns3-kinase) complex is recruited at the reticulum endoplasmic generating curved structures that contain PtdIns3P favoring the recruitment of PtdIns3K complex I, and also facilitating the assembly of PtdIns3P and gathering of WIPI2B, and the E3-like complex (ATG12–ATG5-ATG16L1). The Ptdlns3k complex I consist of phosphatidylinositol 3-phosphate (PtdIns3P), the WIPI proteins; two ubiquitin-like conjugation complexes: (i) one conjugates ATG12 to ATG5 together with ATG16L1 (ATG7, ATG10), and (ii) other that lipidated the Atg8 proteins (ATG7, ATG3, LC3A, GABARAPL1, and GABARAPL2/GATE-16), and ATG9. (iii) Finally, the phagophore is closed; the double-membrane autophagosomes matures and then take place the SNAREs-mediated fusion with lysosomes to degrade its contents (22).

Autophagy has been conceived as a process where intense vesicular trafficking leading to recycling of cytosolic components is the common characteristic, recent evidences showed that transcriptional control of their main molecular players represents also a major regulatory event. Transcription factors TFEB, MiT, and fork-head box members (FOXO) like FOXO1, FOXO3, FOXO4 regulate the expression of diverse autophagy genes including ATG4, ATG12, BECN1, LC3, BNIP3, LC3, ULK1, ULK2, and VPS34 (23, 24). For instance, autophagy flux is controlled by TFEB transcription factor which after phosphorylation is retained in cytosol resulting in inhibition of target genes expression. Conversely, after nutrient starvation the TFEB dephosphorylation causes translocation to the nucleus where it binds to target gene promoters involved in autophagy initiation (*BECN1, WIPI1, ATG9B*, and *NRBF2*) autophagosome membrane elongation (*GABARAP, MAP1LC3B*, and *ATG5*), substrate capture (*SQSTM1*), and autophagosomes trafficking and fusion with lysosomes (*UVRAG, RAB7*). This fine tuning of autophagy genes expression is regulated by mTOR and AKT kinases. In addition p53 transcription factor also controls the expression of key genes for autophagy induction (LKB1, ULK1/2), and autophagosome maturation (ATG4, ATG7, and ATG10) (25).

## Mutations in Autophagy Genes in Endometrial Cancer

In an outstanding paper, Lebovitz and coworkers reported a Pan-cancer study in patient samples and reveal frequent mutations in autophagy genes in endometrial cancer (26). Using data from The Cancer Genome Atlas 211 autophagy-related genes were surveyed for alterations in DNA sequence and mRNA expression. Authors found somatic mutations in a number if autophagy genes including RB1CC1/FIP200, WDR45/WIPI4, ULK4, and ATG7 in endometrial carcinoma and clear cell renal carcinoma. Remarkably, endometrial carcinomas showed a high number of mutations in ATG4C, RB1CC1/FIP200, and ULK4 genes. Also, common mTOR sequence alterations including C1483F and S2215Y hotspot mutations were detected. Moreover, mutations were also observed in endometrioid endometrial tumors including patients that carried out double mutations for PTEN and mTOR genes. Moreover, a truncating mutation (R1321^*^) in RB1CC1 gene was found suggesting autophagy induction may be compromised in patients with type I tumors. In contrast, type II serous tumors exhibited a significant increase of the autophagy inductor CDKN2A.

## Targeting Signaling Pathways Controlling Autophagy in Cancer Endometrial

Diverse approaches to target genes or signaling pathways controlling autophagy are focused in intervention of the autophagic flux. The autophagic flux assays measure autophagic system's degradation activity (27). These methods track the formation and accumulation of autophagosomes, as well as their fusion with lysosomes and the degradation of their content in them. Immunoblotting and immunofluorescence analysis allows the indirect assessment of the number of autophagosomes based on the abundance of MAP1LC3/LC3-II protein. LC3 is known for incorporating into the autophagosome membrane; also a cytosolic form of LC3 (LC3-I) is conjugated to phosphatidylethanolamine to form LC3-phosphatidylethanolamine conjugate (LC3-II), which in turn is recruited to autophagosomal membranes. Other proteins, including p62/SQSTM1 (sequestrosome 1), contain domains that interact with LC3 and serve as adapters between ubiquitinated protein structures and damaged organelles and autophagic machinery. Another important marker of autophagic flux is p62 protein which is located in the autophagosome and is continuously degraded, observing reduced levels when autophagy is activated and an accumulation of p62 when autophagy is inhibited. Immunofluorescence also allows following of autophagy and formation of autophagosomes using antibodies or expression vectors fused to autophagic proteins. An example is vector GFP-LC3; in basal circumstances the GFP-LC3 protein is uniformly distributed in the cytoplasm, however when autophagy is activated, GFP-LC3 is recruited to the phagosomal membrane, generating punctual signals fluorescents in the cell. Finally, transmission electron microscopy (TEM), considered as the gold standard in many autophagy research applications, has the advantage of allowing a direct assessment of autophagosomes in cells (28).

### Autophagy Therapies Related to AKT-mTOR Pathway Inhibition

The aberrant activation of phosphoinositide 3-kinase (PI3K) and the mammalian target of rapamycin (mTOR) signaling occur frequently during the progression of endometrial malignancies. The PI3K/AKT/mTOR signaling is pivotal in regulation of gene transcription, invasion, proliferation, cell survival, and central in the metabolism through regulation of enzymes like Glyoxalase 2 (Glo2) and glyoxalase 1 (Glo1) promoting cancer progression (29, 30). Master regulator mTOR is a serine/threonine kinase with pivotal roles in autophagy that function downstream of PI3K/AKT pathway (31). Under normal cell conditions mTOR impairs autophagy by phosphorylation of mAtg13 and ULK1; whereas in the opposite way after rapamycin stimulation or nutrients starvation, mTOR kinase is repressed (32). Next, we will summarize diverse studies that describe the regulation of autophagic activity through mTOR signaling pathway. Lin et al. (33) reported that oncogene FAM83B expression was significantly higher in endometrial cancer cell lines and tissues relative to normal tissues, and it was associated with myometrial invasion, poor survival, and FIGO II-IV stages (33). Functional analysis showed that FAM83B knockdown leads to the suppression of PI3K/AKT/mTOR pathway, while stimulates autophagy. Furthermore, activation of the pathway turned back the effects of FAM83B silencing-induced autophagy and cancer hallmarks in endometrial cancer cells (33).

In another study, Kanda and colleagues demonstrated that glucagon-like peptide-1 receptor (GLP-1R) agonist liraglutide stimulates autophagy via AMPK pathway in endometrial cancer cells. Authors first showed that treatments with GLP-1R agonist significantly inhibited Ishikawa endometrial cancer cells growth and induced apoptosis. Interestingly, liraglutide also induced autophagy characterized by accumulation of cytoplasmic autophagosomes, higher LC3 expression, decreased p62 levels, and an increased phosphorylated ratio of AMPK kinase (34).

**Figure 1:**
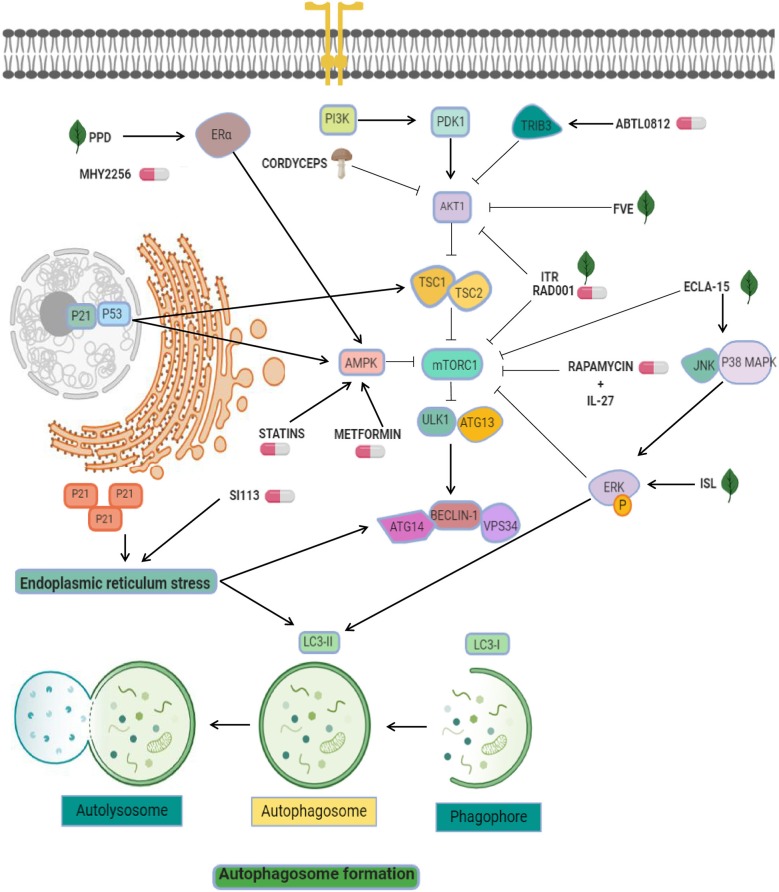
Targeting of pro-autophagy factors in endometrial cancer. Pro-autophagic chemical (pills) or natural compounds (leaf) tested in endometrial cancer cells. AKT inhibitors are used as the main strategy to activate autophagy, and include ABTL0812, FVE, ITR, RAD001, and cordyceps. Other strategies exploit the enhancement of mTOR inhibitors effectiveness through the modulation of AMPK, ERK, and ER-alpha using agents such as ecla-15, ISL, MYH2256, metformin, and statins. Statins and SI113 may induce cytosolic expression of p21, which leads to endoplasmic reticulum stress and autophagy.

On the other hand, functional links between resistance to chemotherapy and autophagy have been dilucidated. Progestins have been used as conservative endocrine treatment in young early endometrial adenocarcinoma patients (35). However, up to 30% of patients showed progestin treatment resistance. Using in-home generated progestin-resistant cells, Liu and coworkers showed that resistant cells acquired increased cell proliferation and resistance to autophagy revealed by a decrease in beclin-1, ATG3, ATG5, and LC3B protein expression relative to parental no resistant cells (36). These changes were associated with PI3K/AKT/mTOR signaling activation. Moreover, treatment of cells with RAD001, a novel mTOR inhibitor, resulted in low mTOR phosphorylation and suppressed the cell proliferation of progestin-resistant endometrial cancer cells by activating autophagy. These data suggested that mTOR can be a therapeutic agent associated to progestin-resistance and autophagy in endometrial cancer (37). In a preclinical study it was reported that RAD001 (known as everolimus), an mTOR inhibitor, suppressed tumor growth and cell proliferation and promoted an additive cytotoxic effect when use together other chemotherapy agents. The underlying mechanisms of RAD001 treatment is based on the inhibition of AKT and mTOR phosphorylation (Figure 1), associated to up-regulation of LC3II protein in Ishikawa (IK) and HEC-1A endometrial cancer cells lines. In addition, an increase in cell death was found after RAD001 treatments in combination with paclitaxel, and interestingly these effects were suppressed after autophagy inhibition (37).

Another effective compound used in endometrial cancer cells is ABTL0812, a fatty acid-derived molecule which may targets the PI3K/AKT/mTOR axis (38). ABTL0812 is an alternative for first line therapy focused in autophagy. Evaluation of its efficacy into IK, AN3CA, and HEC-1A endometrial cancer cell lines showed that ABTL0812 reduced cell viability, promoted the activation of the pro-apoptotic pathway and autophagy induction by TRIB3 overexpression (a negative regulator of AKT1) promoting downregulation of mTOR resulting in autophagy activation (Figure 1) and the transformation of soluble LC3 (LC3-I) to a lipidated form (LC3-II) (38). This molecular mechanism places ABTL0812 as an excellent first-line treatment.

On the other hand, diverse antitumor therapies that have abilities to stimulate autophagy are the combinational use of mTOR inhibitors and proteins related with immune system like interleukins. Natural killer (NK) cells functions are important for sustained tumor growth and therapy response (39) and recently it was shown that both effects exerted by NK cells were enhanced by interleukin (IL)-27. The evidence indicates that IL-27 secreted by endometrial cells may trigger the activation of NK cells in a co-culture system. Related with immune regulation, the Rapamycin is an immunosuppressant and specific inhibitor of mTOR and it has been show that exposure to rapamycin synergistically activates the cytotoxicity of NK cells associated to overexpression of IL-27 (Figure 1). Importantly, this cytotoxicity was favored by the stimulation of rapamycin-mediated autophagy, a signal that was amplified by IL-27, further promoting a suppression of endometrial cancer progression. However, IL-27 could not directly impair cell death and the growth of endometrial cancer cells, but in combination with rapamycin and cisplatin amplifies these effects (40). This knowledge about autophagy activation by mTOR inhibitors and immune system participation is a novel promising direction for endometrial cancer therapies.

Cisplatin (CDDP) and related platinum salts-based molecules are cytotoxic drugs that directly damage the double strand of DNA, inhibiting DNA replication, impairing cellular mitosis and inducing cancer cells death (41). The mechanisms underlying the effects induced by CDDP include the production of reactive oxygen species (ROS), the peroxidation of lipids, activation of p53 signaling, cell cycle arrest, and activation of intrinsic and extrinsic pathways of apoptosis (42). Lin et al. (33) reported that cisplatin modulated the autophagy flux in the endometrial cancer cell line Ishikawa through inhibition of the PI3K/AKT/mTOR signaling. Total and phosphorylated PI3K, AKT, and mTOR proteins were downregulated after treatment with CDDP. Moreover, after cisplatin intervention the number of autophagosomes was augmented relative to untreated controls. Also, the treatment of Ishikawa cells with a PI3K activator, IGF1, partially reversed the effect of CDDP on cell autophagy. These data indicate that conventional cytotoxic therapies may activates autophagy in endometrial cancer cells.

### Autophagy Therapies Related to p53 Pathway

The mismatch repair (MMR) system play a pivotal role in repairing the DNA polymerase errors including diverse types of base mismatches. The MMR machinery activates also cell cycle checkpoints and apoptotic responses following some types of DNA damage including those caused by Sn1-methylating agents N-methyl-N-nitro-N-nitroso guanidine and N-methyl-N-nitrosourea and cisplatin, 5-fluorouracil, temozolomide, and 6-TG (43). The p53 gene is tumor suppressor which plays a key role in safeguarding the genome integrity and it's also an integrator of diverse stressors, such as DNA damage, hypoxia, cell cycle arrest, and programmed cell death (44). After incubation with methylating agents N-methyl-N-nitro-N-nitroso guanidine and N-methyl-N-nitroso urea, the MMR machinery binds to O6-methyl guanine adducts triggering the induction of p53 and apoptosis (45). Zeng et al. (46) analyzed the roles of MMR and p53 signaling in activation of autophagy and apoptosis using MLH1–/MLH1+ colorectal cancer cells (HCT116) as well as MSH2–/MSH2+ endometrial cancer cells (HEC59) after exposure to 6-thioguanine (6-TG). Authors found that MMR repair pathway was required for 6-TG-indiced autophagy, and that p53 tumor suppressor was pivotal for transducing signals from MMR to the autophagic pathway. MSH2 protein was essential for induction of autophagy after 6-TG ^∧^ treatments in endometrial HEC59 (MSH2+, MMR+) cancer cells. Moreover, Atg5 knockdown resulted in enhanced cell death in HCT116 (MLH1+, MMR+) cells following MMR repair of 6-TG damage. In addition, the induction of autophagy resulted in inhibition of apoptosis in response to 6-TG damage, maybe by degradation of damaged mitochondria (46).

## Epigenetic Inhibitors as Modulators of Autophagy in Endometrial Cancer

A link between histone deacetylases inhibitors and mTOR pathway came from a recent study in endometrial stromal sarcomas which are rare tumors representing <1% of all uterine malignancies (47). Histone deacetylases (HDACs) and histone acetyl transferases (HATs) are enzymes that catalyze the modification of histone proteins inducing changes in chromatin structure and function. An increase in histones acetylation in a specific gene promoter results in activation of expression of oncogenes. Therefore, HDACs play pivotal roles in the development of diverse human tumors; thus HDAC targeting with inhibitors represents a promising approach in cancer therapies (48). Another HDAC inhibitor is the suberoylanilide hydroxamic acid (SAHA) which blocks HDACs activity inducing cell differentiation, cell cycle arrest, and cell death of cancer cells (49). In ESS-1 cells, the suppression of mTOR by SAHA leads to impairment in cell proliferation (50). Recent data showed that increased phosphorylation of the S6 ribosomal protein S6rp was involved in autophagy activation. Phosphorylation of p70S6K kinase (Ser235/236) which in turns phosphorylates S6rp regulating in this way the synthesis of proteins involved in cell growth and cell cycle progression in ESS-1 cells but not in HESCs (51). ESS-1 cells were more sensitive to SAHA inhibitor than normal endometrial stromal cells, and significantly inhibited the proliferation of ESS-1 cells by inducing cell cycle arrest and activating autophagic process. The data suggested that activation of autophagy by modulating HDACs activity could have clinical potential in treatment of uterine and endometrial sarcomas (52).

On the other hand, recent evidence has implicated p53 as an unexpected player in autophagy regulation via apoptosis activation (53). Several studies have shown that HDACs inhibitors have a potential clinical value in endometrial cancer therapy. For instance, it was showed that MHY2256, an inhibitor of sirtuin (SIRT) protein, has antitumor effects inducing the acetylation of p53 protein in estrogen positive breast cancer cells (54). Further, the same group reported that MHY2256 treatment in endometrial cancer cells enhanced late apoptosis and significantly reduced tumor growth in a mouse xenograft model (55). The anticancer activity autophagy-associated in Ishikawa cells was observed at low concentrations of MHY2256 leading to increases of LC3-II and autophagy-related protein 5 (ATG5), furthermore MYH2256 significantly increased the red florescence acidic vesicular organelles (55). There is a possibility that the mechanism of autophagy activation was related to cell cycle regulators p21 and acetylated p53 (Figure 1). Some reports highlight this possibility. For example, it was reviewed that p53 mediated autophagy-regulation by increased of tuberous sclerosis complex 2 (TSC2) and AMPK, resulting in suppressing mTOR and the activating ULK1 complex (53). Also, p21 induced autophagy and senescence in breast cancer (56), and quinacrine showed antitumor effects by inducing p21-dependent autophagy in HCT-116 colorectal cancer cells (57). These data suggested that Sirtuin inhibitors may modulates autophagy and could be an interesting therapeutic tool in cancer.

## Endoplasmic Reticulum-Related Therapies in Endometrial Cancer

Endoplasmic reticulum (ER)-stress is a powerful trigger of autophagy. It has been reported that ER stress leads to upregulation of genes related to autophagy activation, including ATG8, ATG14, and facilitate the formation of the phagophore and autophagosomes through ER-membrane bound ATG9 (58). Interestingly, some therapeutic molecules have been shown to activate autophagy through this pathway in endometrial cancer cells. For instance, the serine/threonine protein kinase SGK1 showed anticancer activity because its ability to controls oxidative and ER stresses. SI113 compound has been recently identified as a potent and selective inhibitor SGK1 and AKT kinases activity with the ability to trigger the autophagic process. In particular, treatment with SI113 in endometrial cancer cells promoted the increase in LC3B-II and beclin1 levels (59). Activation of autophagy appears to be connected with induction of apoptosis and the cleavage of both PARP and caspase-9 proteins. Furthermore, these effects were related to the activation of ER stress GRP78 and CHOP proteins (Figure 1). These promising effects place the inhibitor as a promising therapeutic approach in endometrial cancer (59).

## Autophagy Modulation Using Repurposing Compounds in Endometrial Cancer Therapy

Drug repurposing (also referred to as repositioning or redirection) refers to the utilization of current therapeutic medications originally developed for one specific health condition to treat alternative indications such as cancer, which leads to easy access of these agents for patients. In addition, the utilization of these drugs reduces the cost associated to development of novel oncologic agents (60). For instance, epidemiologic studies indicated that dimethylbiguanide metformin, an orally administered medication commonly used to maintain low blood glucose in individuals with non-insulin-dependent diabetes mellitus, have also significant chemopreventive effects by decreasing the risk to develop diverse human cancers including colon, pancreatic, breast, and prostate (61), and recent evidence shows that it is also able to stimulate autophagy based on activation of AMP-activated protein kinase (AMPK). On this topic, three reports described the up-regulation of AMPK, p-AMPK, LC3II, and beclin1 after metformin treatment in endometrial cancer cells (62–64). AMPK is an important factor involved in the inhibition of tumor growth and autophagy triggering. Two mechanisms have been proposed for modulation of autophagy: (i) the mTOR inhibition by AMPK, and (ii) the activation of ULK1 to induce autophagic processes (65). Another investigations combined metformin with natural agents such as ginseng saponin protopanaxadiol (PPD) resulting in enhanced anti-tumor effects induced by metformin and unexpectedly reducing the levels of estrogen receptor alpha. It has been shown that estrogen stimulated the cell viability and blocks the cell death and autophagy of Ishikawa and RL95-2 cells, and that combinations of protopanaxadiol and metformin effectively revers the aforementioned cellular effects (64).

**Figure 2:**
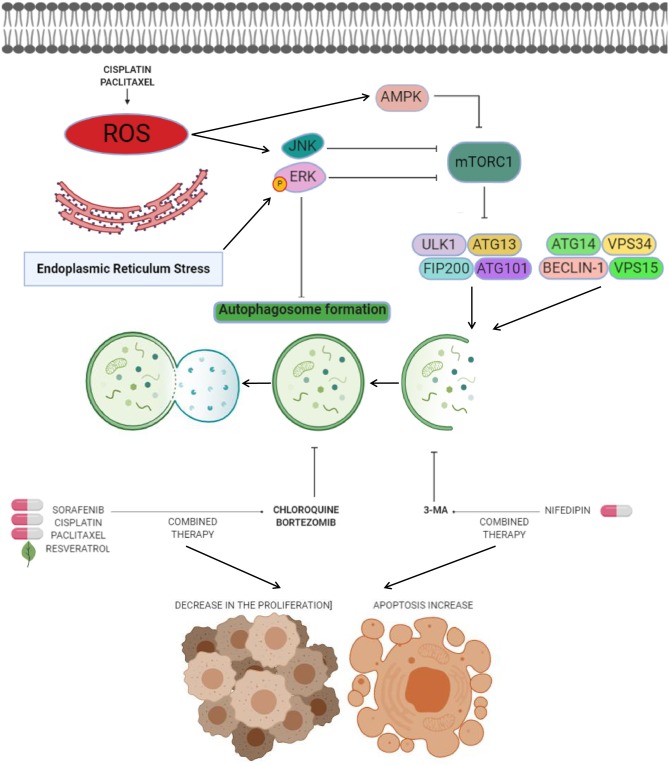
Anti-autophagic approaches in endometrial cancer. Anti-autophagic chemicals (bold letters), autophagy inducing chemicals (pills), or natural compounds (leaf) tested in endometrial cancer cells. The production of reactive oxygen species (ROS) after cisplatin and paclitaxel therapies mediates the autophagy process. ROS have been shown to induce autophagy by several processes including activation of ERK and JNK transducers, as well as inhibition of mTOR signaling by AMPK. Novel strategies for therapeutic intervention in endometrial cancer are based on the suppression of autophagy through use of agents such as CQ or 3-MA to promotes cell death and decreases in cell proliferation when combine with agents like sorafenib, cisplatin, paclitaxel, resveratrol, and nifedipin. Other agents such as bortezomib block cisplatin-induced autophagy and stimulate the cytotoxic effects of chemotherapy on cancer cells.

Chloroquine is a therapeutic molecule widely used to treat malaria disease. It's now known that chloroquine inhibits autophagy by a mechanisms associated to the increase of lysosomal pH, and its antitumor effects have been documented in brain, breast, lung, and colon cancers (66) as well as in endometrial cancer cells. Interestingly, chloroquine also has been reported to exert anticancer effects through autophagy-independent mechanisms such as lysosomal accumulation, mitochondrial disintegration, selective necrosis of tumoral cells, normalizing tumor vasculature, and reducing tumor hypoxia, causing the cancer cell growth inhibition, cell death, and an increase in the therapy responses (67, 68). Direct effects of chloroquine in autophagy have been reported by Fukuda and coworkers which showed that chloroquine intervention of resistant endometrial cancer cells inhibited autophagy and partially restored its sensitivity to cisplatin (69). The suppression of autophagy using chloroquine increased both cisplatin and paclitaxel-induced cell death in HEC-1A and JEC endometrial cancer cells; furthermore the sensitivity to cisplatin was increased after chloroquine intervention. As previously observed, ROS generation following the treatment with these drugs mediate the autophagic process by activating of ERK, AMPK, and JNK signaling, and impairs mTOR pathway (Figure 2) (69). These data highlight the chemosensitization effects of chloroquine associated to autophagy inhibition in endometrial cancer cells.

One of the most attractive approaches in oncologic treatments is the combination of autophagy inhibitors such as sorafenib with agents like chloroquine to synergize their antitumor effects. Sorafenib is a FDA-approved broad-spectrum kinase inhibitor which was initially used as an inhibitor of RAF1 but it also has inhibitory effects on BRAF kinase (70). Sorafenib is commonly used in patients with renal cell and hepatocellular carcinoma, and recently it has been suggested as therapeutic in endometrial carcinoma (3, 71). However, increased resistance to sorafebib limited its clinical utilization in patients. Eritja and coworkers demonstrated that sorafenib resistance was associated to autophagy endometrial cancer cells. This compound activates a MAPK/JNK-dependent protective autophagic mechanism in endometrial cancer after therapeutic stress (3). The evidence supported that sorafenib exposure in endometrial cancer cells promoted the modification of LC3B- I to LC3B-II, which was accompanied by apparition of autophagic structures. However, in conditions of autophagy, the silencing of Beclin 1 using siRNAs and chloroquine sensitized endometrial cancer cells to sorafenib treatment (Figure 2). *In vivo* studies showed that targeting autophagy resulted in enhanced sorafenib cytotoxicity and suppressed tumor growth and pulmonary metastasis. These results grant novel insights about the role of sorafenib in the activation of a protective autophagic response as a new strategy for therapeutic intervention in endometrial cancer (3).

Another valuable approach related to the inhibition of autophagy as a therapy in endometrial cancer is the use of bortezomib in combination with platinum-based chemotherapy. Bortezomib is a novel inhibitor of the 26S proteasome, which exhibit anticancer properties in diverse types of human neoplasias including colon, breast, ovarian, and prostate cancer (72–74). Its molecular mechanism focuses on the inhibition NF-κB pathway resulting in augmented sensitivity of cells to chemotherapy via apoptosis activation (65). It has been shown that sustained activation of ERK may inhibit the autophagy process (Figures 1, 2) (75, 76). In this context it could be explained that bortezomib inhibits the fusion of lysosome and autophagosome promoting p62 accumulation at the autophago-lysomal stage in endometrial cancer Ishikawa cells. Interestingly platinum-based chemotherapy activates autophagy in ovarian cancer cells resistant to cisplatin and bortezomib block the cisplatin induced autophagy stimulating the chemotherapy efficacy in ovarian cancer (77).

Anti-autophagic approaches using repurposing compounds are also related to arterial hypertension medications such as nifedipine, an L-type calcium channel antagonist that suppress the cell proliferation of diverse types of cancer (78). The effect of nifedipine on HEC-1A endometrial cancer cells was the suppression of cell proliferation and triggering of apoptosis. Furthermore, Nifedipine also induced autophagy and staining analysis revealed that the formation of autophagic GFP-LC3-II was stimulated by nifedipine treatment. Interestingly, the autophagy inhibitor 3-MA combined with nifedipine activated cell death indicating that autophagy may promotes the cell survival associated to Beclin1 and mTOR functions (79).

The combination of chloroquine and paclitaxel (Taxol) has become a promising strategy. Paclitaxel acts by binding to β-tubulin thereby inhibiting microtubule depolymerization in cytoskeleton, and consequently resulting in cell cycle arrest at G2/M stage and cell death (80). Surprisingly, it has also been shown to be an autophagy activator in diverse types of cancers (81, 82). Paclitaxel exposure in endometrial cancer HEC-1A and JEC cells induce autophagy-related events such as augmented LC3-II/LC3-I ratio and low p62 abundance (83). Perhaps the mechanism of paclitaxel-induced autophagy is related with the generation of intracellular ROS. In previous studies, it has been described that anticancer agents can promote the generation of ROS and in turns activates autophagy after turning on ERK, JNK, and AMPK transducers (Figure 2) (83, 84). An interesting effect in sensitivity of endometrial carcinoma cells to chemotherapy was observed when autophagy was inactivated through knockdown of Beclin 1 and by treatment with chloroquine. Also, combined intervention with chloroquine and paclitaxel leads to autophagy abrogation and high proportion of HEC-1A and JEC cell death (83). These findings suggested that approaches based on the inhibition of autophagy can open new paths to improve the paclitaxel efficiency in endometrial cancer therapy.

## Autophagy and Chemotherapy Resistance

Novel clues about the relationships between the cellular mechanisms underlying drug resistance, stemness, and autophagy in endometrial cancer were provided in a study by Ran et al. (85). Using CD133+/CD44+ cancer stem cells-like isolated from the JEC endometrial cancer cell line they found an increase in autophagy relative to parental endometrial cells. Moreover, autophagy inhibition was associated with the inhibition of stem cells-like phenotype, specifically diminished spheroids formation and enhanced sensitivity to paclitaxel. These data support the notion that stemness phenotype and activation of pro-survival autophagy are both related to chemoresistance of cancer stem-cells. In addition it was found that the estrogen induced gene (EIG121) promoted both autophagy and cell survival in the subpopulation of CD133+/CD44+ cells and normal endometrial cancer cells (85).

## Targeting Autophagy With Natural Dietary Compounds

Several natural compounds exhibit promising effectiveness against endometrial cancer cells in *in vitro* studies, and therefore have been proposed as attractive therapeutic agents in aggressive endometrial cancers. These compounds have a direct impact on several metabolic pathways, as the autophagy, but their role in cancer is contradictory due it has a dual function in survival and cell death. Nevertheless, autophagy targeting can be exploited as a new therapeutic target to contribute with development of alternative and more effective treatments in endometrial cancer. Natural compounds provide opportunities for combinatorial therapies which may affects at the same time multiple targets to achieve a higher effectiveness in comparison to that of single molecule-based drugs.

Hedgehog signaling pathway inhibitors have been shown to successfully impair proliferation of endometrial cancer cells (86), and mTOR inhibitors have also been investigated for their therapeutic potential in endometrial cancers (87). Itraconazole is a common antifungal agent that inhibited Hedgehog and AKT/mTOR signaling transducers as well as WNT/β-catenin signaling (88) and showed a dose- and time-dependent suppression of cell proliferation in human endometrial cancer cell lines (89). Tsubamoto and coworkers reported that this fungal agent also suppressed the proliferation of AN3-CA, HEC-1A, and Ishikawa cells, but did not suppress GLI1 or GLI2 transcription, downstream effectors of the Hedgehog pathway in HEC-50B or SNG-II cells (90). Moreover, itraconazole also inhibited the expression of signaling proteins in HEC-1A and AN3-CA cells, and upregulated the microtubule-associated protein 1A/1B-light chain 3-II. In Ishikawa, HEC-50B and SNG-II cells, the ATP-binding cassette transporter A1 (ABCA1) protein expression was suppressed following itraconazole treatment (90). ABCA1 regulates cholesterol efflux across the plasma membrane. Itraconazole treatment also appears to inhibit the growth of cancer cells by blocking the activation of AKT/mTOR signaling. In addition, the consistent activation of AKT was associated with higher rates of lipid raft formation, while its abrogation impeded AKT activation, and the intracellular cholesterol trafficking to the plasma membrane in human umbilical vein endothelial cells, thereby suppressing mTOR (91).

The isoliquiritigenin (ISL) is a licorice flavonoid with anti-oxidant, anti-inflammatory, and tumor suppression effects. In telomerase-immortalized endometrial stromal (T-HESCs), Ishikawa, HEC-1A, and RL95-2 cells the treatment with ISL resulted in a reduction of cell growth and survival in a dose- and time-dependent way (92). ISL also inhibited the proliferation and induced the arrest of cell cycle (G1 and G2/M phase) through activation of p53/p21 signaling. It also promoted apoptosis associated with the activation of caspase-3, caspase-7, and PARP, and promoted the autophagy by an accumulation of LC3II protein and higher levels of phosphorylated p-ERK. The ERK-dependent autophagic activity was associated with the LC3 induction and the conversion of LC3I to LC3II (93, 94). ISL also suppressed the growth of HEC-1A-LUC xenograft tumors and suppressed the expression of nuclear PCNA. These effects were accompanied by increased caspase-7, p62, and LC3B protein expression in tumor tissues suggesting that it could be a potential anti-cancer drug candidate (95).

Resveratrol is a polyphenolic compound derived from red wine and red fruits with well-documented anti-tumor effects. Also it exhibits the ability to enhance autophagy by an increase of LC3-II accumulation, and suppressed the proliferation of Ishikawa cells in lower dose (20 μM) than other cancer cell (96). Resveratrol treatment also increased the expression of p-AMPKα that has been associated with its ability to induce apoptosis (97), and repressed autophagy by the inhibition of mTOR signaling pathway (98, 99). The activation of AMPK by resveratrol also counteracts the inhibition of the mTOR-dependent autophagy (thereby resulting in autophagy promotion). The combinatory treatment of resveratrol with chloroquine markedly suppressed the cell growth and suppressed apoptosis, compared with the resveratrol treatment alone. The inhibition of autophagy process after silencing of ATG5 or ATG7 genes using siRNAs effectively activated the cell death in endometrial cancer cells (Figure 1).

*Fucus vesiculosus* (brown seaweed) extracts (FVE) appears to have display anti-cancer effects in estrogen receptor (ER)-dependent and -independent way in HEC-1-B and RL95-2 endometrial cancer cell lines by a competitive inhibition of estradiol (E2) binding to the estrogen receptor (100, 101). FVE inhibited aromatase enzyme activity *in vitro* and a co-treatment with E2 reduced the estrogen receptor activation by 50%, inhibited endogenous E2 and significantly decreased viability of cells. FVE also induced the expression of apoptotic (CASP6; APAF1, FANCG, XIAP, MED1), autophagy (ATG10, GABARAP) proteins, and BRAF, PIK3R4, PRKAA1, PRKACB, PRKAR1A, PRKAR2A, and MAP3K14 kinases. In addition altered morphological features in the cells suggested active apoptosis and autophagy, evidencing the effects of FVE as an autophagic-mediator of apoptosis, associated to low phosphorylation of proteins from PI3K/Akt/mTOR signaling (Figure 1). It has been reported that FVE may also be effective in the therapy of breast, ovarian, and endometrial cancers. These findings suggested that FVE may achieve a protective effect against estrogen-dependent cancers (102).

Triterpenoids echinocystic acid and its glycosides isolated from *Eclipta prostrata* exhibits diverse protective activities in malaria, HIV, and cancer diseases. They also showed anti-venom, antioxidant and anti-inflammatory abilities (103–106). Isolate compounds have cytotoxic effects in endometrial cancer cells, in that way, eclalbasaponin II (one glucose moiety) > echinocystic acid >> eclalbasaponin I (two glucose moiety) suggesting that the cytotoxic activity of oleanane-type triterpenoids was associated with the sugar moiety at the C-3 position and the free carboxyl at C-28 (107). Eclalbasaponin II treatment induced apoptosis in a caspase-independent manner (type II programmed cell death) in ovarian cancer cells by an increase in the sub G1 population. Also, it was found an increase in acidic vesicular organelle content and an in the levels of LC3-II. Moreover, eclalbasaponin II also activated MAP kinase JNK and p38 proteins and repressed the mTOR pathway (Figure 1). Therefore, these perennial herb-derivate compounds exhibit anti- tumor activities in ovarian and endometrial cancer cells (108).

Three fungi *Cordyceps Sinensis* (Cordy)*, Ganoderma lucidum* (Reishi), and *Agaricus Blazi Murill* (ABM) extracts have biological activities and numerous pharmacological effects and are commonly utilized in traditional Chinese medicine as adjuvant in cancer therapies (109–111). Cordy and Reishi extracts has been also used in leukemia, ovarian, breast, prostate, and gastric cancer (112–115). Crude extracts derived from Cordy and ABM/Reishi had an inhibitory effect on cell viability, proliferation, and suppression of cell growth of Ishikawa, Hec-1A, and AN3-CA cells by suppression of p-AKT (116). Remarkably, Beclin-1 phosphorylation by p-AKT was essential for autophagy and anchorage-independent growth (117). The low levels of p-AKT lead to higher autophagy and endometrial cancer cells death. In tumors, the p-AKT level PI3K/AKT pathway has been associated with cisplatin-resistance (118) and unlike other fungi, Cordy and ABM/Reishi extracts did not affects the interplay between endometrial cancer cells and NK-cells *in vitro* (Figure 1).

## Non-coding RNAs Regulating Autophagy

Recent studies strongly suggested the participation of microRNAs (miRNAs) in the progression of endometrial cancer, at least in part, through autophagy modulation. MiRNAs are small non-coding RNAs of 25 nucleotides length. These tiny RNAs function as master regulators of gene expression at posttranscriptional level by blocking translation and activating the degradation of specific transcripts or mRNAs in cellular cytoplasmic P-bodies (119). Alterations in the miRNAs abundance have been found during early and late stages of endometrial cancers. For example, Wang and Liu demonstrated that miR-101-3p activates the autophagy in endometrial cancer cells by binding and subsequent downregulation of EZH2 mRNA (120). Some miRNAs, such as miR-218 and miR-205, have been demonstrated to modulate chemoresistance in endometrial cancer (121, 122). Particularly, miR-218 was downregulated in paclitaxel-resistant endometrial cancer cells relative to non-resistant parental cells, whereas upregulation of miR-218 leads to sensitization of paclitaxel-resistant endometrial cancer cells to paclitaxel. In addition, miR-218 repressed autophagy by targeting HMGB1 in paclitaxel-resistant cells (121). On the other hand, Zhuo and colleagues demonstrated that miR-205 was upregulated in a progesterone-resistant (PR) sub-cell line and induced apoptosis through repressing autophagy process by targeting tumor suppressor PTEN through AKT/mTOR signaling in endometrial cancer (122). Finally, a recent study showed the participation of the HOX Transcript Antisense RNA (HOTAIR) in the regulation of the resistance to cisplatin in endometrial cancer cells. HOTAIR controlled autophagy by modulating the expression of Beclin-1, multidrug resistance (MDR), and P-glycoprotein (PGP) proteins (123). Thus, these initial reports showed that miRNAs and long non-coding RNAs may participate in the process of endometrial tumorigenesis and development by influencing autophagy.

## Conclusions

Various roles of autophagy have emerged in the scientific literature and the relationship between autophagy and cancer is enormously controversial due to its dual roles depending on the context and tumor environment. From the biological view of cancer, even particularly in endometrial cancer, it remains controversial whether autophagy is a tumor suppressor process (by stopping the cell cycle, activating apoptosis, decreasing proliferation) or oncogenic process (promoting cell survival against insults caused by chemotherapy agents). Endometrial cancer is the fourth most frequent neoplasia for women worldwide. Combinatory therapy with carboplatin and paclitaxel is the main front-line chemotherapy in endometrial cancer. However, its clinical effectiveness is variable, indicating that novel molecular therapies against specific cellular processes associated to cell survival and therapy resistance urged to ameliorate the rates of success in endometrial cancer therapies. Autophagy represents in this context a potential cellular target for the development of new therapeutic agents. Although the knowledge of autophagy mechanisms in endometrial cancer are limited, important recent findings in cell lines and patients greatly help us to visualize the potential intervention of these human carcinomas in order to ameliorate the rates of successful therapy for patients. As we have reviewed in this work, various signaling pathways such as PI3K-AKT-mTOR, AMPK-mTOR, and p53 trigger or inhibit the autophagy process and can be used as potential molecular targets in therapeutic approaches in two ways: (i) inhibiting autophagy to promote sensitization of endometrial tumor cells in response to chemotherapy agents such as cisplatin and paclitaxel, and (ii) its activation in some endometrial cancer cell lines is related with low cell proliferation, migration, invasion, and activation of apoptosis. However, despite the discrepancies in these studies, we believe that variables such as tumor stage, alteration in the balance of cell signaling, epigenetics, cell cycle, and even mutations in autophagy genes could be playing important roles that lead the results of the investigations at both ends: oncogenic and tumor suppressor. Of interest are the findings that autophagy can be modulated using repurposing compounds which may leads to faster experimentation and validation, and access of these drugs to patients. Hence, although the research about autophagy is ongoing, the actual studies highlight the potential usefulness of novel molecules and proteins for directed targeted therapies in endometrial cancer.

## Author Contributions

SN-O, DG-R, JP-R, YS-V, LM, RM-V, and CL-C wrote all the manuscript sections. SN-O and JP-R draw the figures. DG-R, LM, and CL-C conceived and designed the review.

### Conflict of Interest

The authors declare that the research was conducted in the absence of any commercial or financial relationships that could be construed as a potential conflict of interest. The handling Editor declared a past co-authorship with one of the authors, CL-C.
